# Artemisinin Derivatives Stimulate DR5-Specific TRAIL-Induced Apoptosis by Regulating Wildtype P53

**DOI:** 10.3390/cancers12092514

**Published:** 2020-09-04

**Authors:** Xinyu Zhou, Sietske N. Zijlstra, Abel Soto-Gamez, Rita Setroikromo, Wim J. Quax

**Affiliations:** Department of Chemical and Pharmaceutical Biology, Groningen Research Institute of Pharmacy, University of Groningen, Antonius Deusinglaan 1, 9713 AV Groningen, The Netherlands; xinyu.zhou@rug.nl (X.Z.); s.n.zijlstra@student.rug.nl (S.N.Z.); a.a.soto.gamez@rug.nl (A.S.-G.); R.Setroikromo@rug.nl (R.S.)

**Keywords:** artemisinin, TRAIL, death receptors, colon cancer, 3D spheroid

## Abstract

**Simple Summary:**

The risk of developing colorectal cancer at a younger age has increased, but current therapies are either risky or limited. We aim to demonstrate that the combination treatment of artemisinin derivatives and TRAIL could be a potential therapy to kill colon cancer cells. We found that artemisinin derivatives increase death receptor production and further sensitize colon cancer cells to TRAIL-induced apoptosis. Furthermore, we explored the role of P53 in response to artemisinin derivatives, which transactivates Death Receptor 5 (DR5) and the cyclin-dependent kinase inhibitor P21. Finally, a 3D tumor spheroid model also confirmed the efficacy of the combination treatment.

**Abstract:**

Artemisinin derivatives, widely known as commercial anti-malaria drugs, may also have huge potential in treating cancer cells. It has been reported that artemisinin derivatives can overcome resistance to tumor necrosis factor-related apoptosis-inducing ligand (TRAIL)-induced apoptosis in liver and cervical cancer cells. In our study, we demonstrated that artesunate (ATS) and dihydroartemisinin (DHA) are more efficient in killing colon cancer cells compared to artemisinin (ART). ATS/DHA induces the expression of DR5 in a P53 dependent manner in HCT116 and DLD-1 cells. Both ATS and DHA overcome the resistance to DHER-induced apoptosis in HCT116, mainly through upregulating death receptor 5 (DR5). We also demonstrate that DHA sensitizes HCT116 cells to DHER-induced apoptosis via P53 regulated DR5 expression in P53 knockdown assays. Nevertheless, a lower effect was observed in DLD-1 cells, which has a single Ser^241^Phe mutation in the P53 DNA binding domain. Thus, the status of P53 could be one of the determinants of TRAIL resistance in some cancer cells. Finally, the combination treatment of DHA and the TRAIL variant DHER increases cell death in 3D colon cancer spheroid models, which shows its potential as a novel therapy.

## 1. Introduction

Artemisinin (ART) is a sesquiterpene lactone extracted from the sweet wormwood (*Artemisia annua*) and approved as an anti-malaria drug [1,2]. The causative parasite for malaria, *Plasmodium falciparum*, degrades hemoglobin to produce amino acids and liberate heme-iron in the red blood cell [3]. When treated with artemisinin, the iron(II) oxide cleaves the endoperoxide bond of artemisinin to generate highly reactive carbon-based free radicals to kill *P. falciparum.* The rich source of intracellular Fe^2+^ from hemoglobin in red blood cells is an essential component of the mechanism for artemisinin activation [3,4]. Compared to healthy cells, cancer cells contain high intracellular iron content to maintain proliferation and high metabolic activity, which makes them susceptible to artemisinin via the same mechanism as described above [5,6]. In cancer cells, the formed radicals are involved in protein alkylation and reactive oxygen species (ROS) production, which trigger DNA damage, induce apoptosis and ferroptosis, and reduce proliferation [7,8].

Tumor necrosis factor-related apoptosis-inducing ligand (TRAIL) mainly induces apoptosis in transformed cells, leaving healthy cells intact, which makes it a potential cancer therapy [9,10]. TRAIL induces cell apoptosis via binding with death receptor 4 (DR4) or death receptor 5 (DR5). This function is antagonized by binding to decoy receptor 1 (DcR1), decoy receptor 2 (DcR2), or soluble decoy receptor osteoprotegerin (OPG) [11,12]. Two signaling pathways have been described to initiate the apoptosis program in mammalian cells: intrinsic and extrinsic apoptotic pathways. The extrinsic pathway can be initiated when TRAIL binds to one of the two death receptors forming the death-inducing signaling complex (DISC), which will lead to the recruitment of the Fas-associated death domain (FADD), followed by the activation of the caspase cascade. For the intrinsic pathway, activated caspases cleave BH3 interacting death domain (BID) to truncated BID (tBID), which will activate Bax and Bak to stimulate the release of cytochrome C from mitochondria. Cytochrome C forms the apoptosome with apoptotic protease activation factor 1 (APAF-1) to cleave pro-caspase-9, which, in turn, activates caspase-3. Both intrinsic and extrinsic pathways lead to the activation of caspase-3, which eventually leads to cell apoptosis [13,14]. 

Colorectal cancer, as the third most commonly diagnosed tumor disease in men and women, is also the second cause of cancer-related deaths [15]. Although the death rate from colorectal cancer (CRC) is decreasing, the risk of developing CRC at younger ages has increased [16]. Therefore, there is an urgent need to develop a promising therapeutic method and to explore the inter-individual differences in colon cancer. However, the resistance of colorectal cancer cells to TRAIL-induced apoptosis prohibits the option of TRAIL to become an effective clinical therapy.

**Figure 1:**
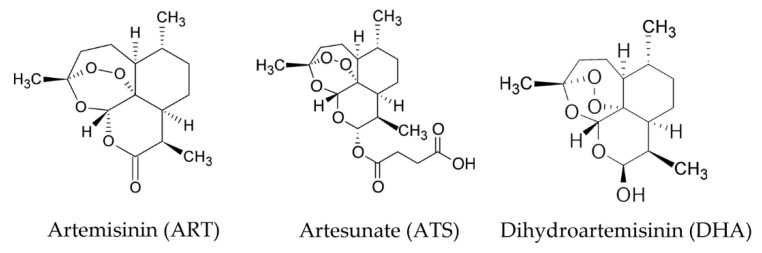
Chemical structure of artemisinin (ART), artesunate (ATS), and dihydroartemisinin (DHA).

It has been reported that artemisinin derivatives can overcome TRAIL resistance in different cancer cell lines [17,18,19,20]. Artesunate (ATS) and dihydroartemisinin (DHA) are two derivatives of ART, which show higher efficacy, solubility, and bioavailability compared with ART in malaria [3,21,22] (Figure 1). These drugs can be taken by oral administration, intravenously, or by intramuscular injections for treatment [3,23]. However, ATS is the only compound that allows for all types of administration due to its better solubility [24]. Meanwhile, in the metabolic process, ATS is first converted to DHA to execute its functions, which makes DHA a promising candidate in treating cancer cells [25].

Here, we utilized the artemisinin derivatives in combination with the DR4-specific TRAIL variant 4C7 [26] and the DR5-specific TRAIL variant DHER [27] to stimulate TRAIL-induced apoptosis in colon cancer cells. We also examined the influence of ATS and DHA on DR4 and DR5, in terms of gene transcription, protein level, and cell surface expression. Finally, artemisinin derivatives were found to significantly enhance cell death in combination with DHER in 3D colon cancer cell spheroids. 

## 2. Results

### 2.1. Artemisinin Derivatives Sensitize Colon Carcinoma Cell Lines to TRAIL-Induced Apoptosis

**Figure 2:**
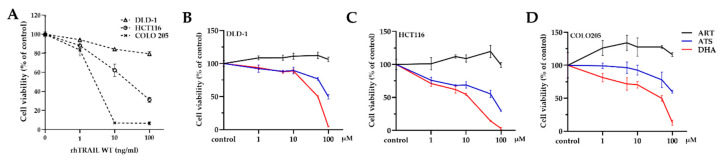
Sensitivity of colon carcinoma cell lines to artemisinin derivatives and rhTRAIL WT treatment. (**A**) The sensitivity of different colon cancer cells to TRAIL-induced apoptosis; all the cell lines were treated with 0, 1, 10, 100 ng/mL of rhTRAIL WT for 24 h. DLD-1 (**B**), HCT116 (**C**), COLO 205 (**D**) were treated with 0, 1, 5, 10, 50, 100 μM of ART, ATS/DHA for 24 h. Cell viability was determined by MTS assay. Data shown are mean ± SD from one of at least two independent experiments performed in triplicate.

Dose-dependent reduction of cell viability by wild type (WT) recombinant human TRAIL (rhTRAIL) in colon carcinoma cells was analyzed using [3-(4,5-dimethylthiazol-2-yl)-5-3(carboxymethoxyphenyl0-2-(4-sulfophenyl)-2H-tetrazolium] (MTS) assay. COLO 205 is highly sensitive; HCT116 is sensitive; DLD-1 is less sensitive to TRAIL-induced apoptosis (Figure 2A). Moreover, the cells were exposed to different concentrations (0, 1, 5, 10, 50, 100 μM) of ART, ATS, or DHA for 24 h. The data showed that all these cell lines are resistant to ART (<100 μM) but sensitive to both ATS and DHA at the highest concentration (Figure 2B–D). This might be due to their better solubility.

**Figure 3:**
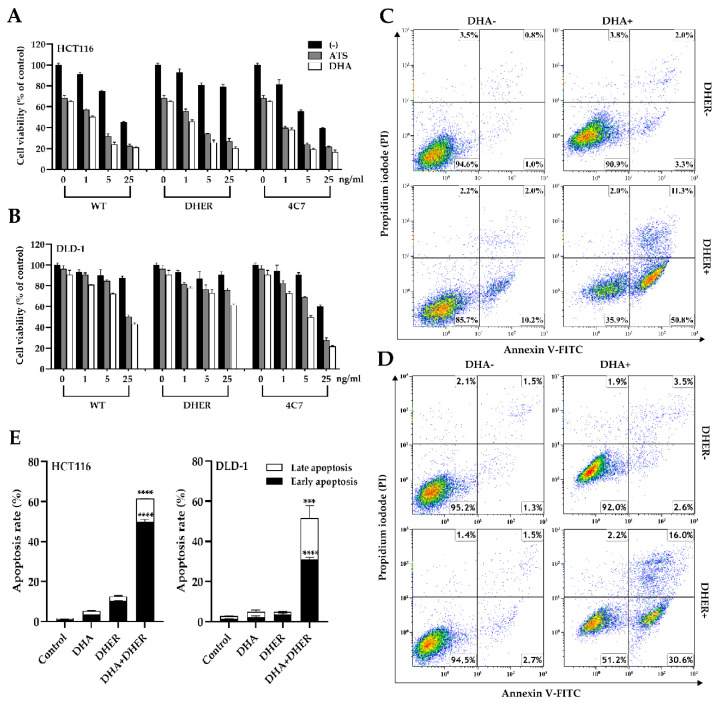
Artemisinin derivatives influence the efficacy of rhTRAIL WT, DHER, or 4C7 in colon cancer cells. HCT116 (**A**) and DLD-1 (**B**) were pretreated with 10 μM ATS/DHA for 30 min. Then, they were all incubated with different concentrations of rhTRAIL WT, DHER, or 4C7 for 24 h. Cell viability was determined by MTS assay. Data shown are mean ± SD from one of three independent experiments performed in triplicate. (**C**) HCT116 and DLD-1 (**D**) were treated with 10 μM DHA for 30 min, followed with/without 25 ng/mL DHER for 16 h. Statistical analysis was shown in (**E**). Apoptosis assay was performed by flow cytometry using annexin V/PI staining, and data were analyzed by Kaluza 2.1.1. *p* values were analyzed by two-way ANOVA with Dunnett’s multiple comparisons test in Graphpad Prism version 8.0. *** 0.0001 < *p* ≤ 0.001, **** *p* ≤ 0.0001.

Then, we analyzed the effect of ATS and DHA with the combination of DR5-specific TRAIL variant DHER and DR4-specific TRAIL variant 4C7 in the mentioned colon cancer cell lines (except for COLO 205, which is already highly sensitive to TRAIL). Both HCT116 and DLD-1 are sensitive to the cell death induced by 4C7 but resistant to DHER (Figure 3A,B, black bar), which corresponds to our previous report [28]. The cell viability observed in the single treatment with ATS (grey bar) or DHA (white bar) is ~70% for HCT116 and ~90% for DLD-1, respectively. In HCT116, cell death increases with increasing concentrations of rhTRAIL WT or 4C7, and the same trend occurs with the additional ATS/DHA treatment. Interestingly, 25 ng/mL of DHER on its own leads to around 20% cell death, whereas, with the combination of ATS/DHA, 80% of the cells die (*p* < 0.0001). In addition, DHER shows dose-dependent cell death in the presence of ATS/DHA, which indicates that artemisinin derivatives overcome DHER resistance in HCT116 cells (Figure 3A). Moreover, ATS/DHA shows partial influence when combined with DHER but significantly enhances the efficacy of WT and 4C7 in DLD-1 (Figure 3B) (*p* < 0.0001). 

To confirm the association of DHER-mediated cell death with apoptosis, we performed an annexin V/propidium iodide (PI) apoptosis assay. As shown in Figure 3C,D, the number of early apoptotic cells in the combination treatment is significantly higher compared to DHA or DHER individual treatment in HCT116 and DLD-1. Specifically, 61% of cells become apoptotic cells in the combination of DHA and DHER, while this occurs for only 5% or 12% in the case of individual treatment in HCT116. The same phenomenon was observed in DLD-1; the combination treatment induces 51% of cell apoptosis, which is much higher than the individual treatments (5% in DHA, 5% in DHER) (Figure 3E). 

### 2.2. DHA Enhances TRAIL-Induced Apoptosis through Caspase-Mediated Death Signal

**Figure 4:**
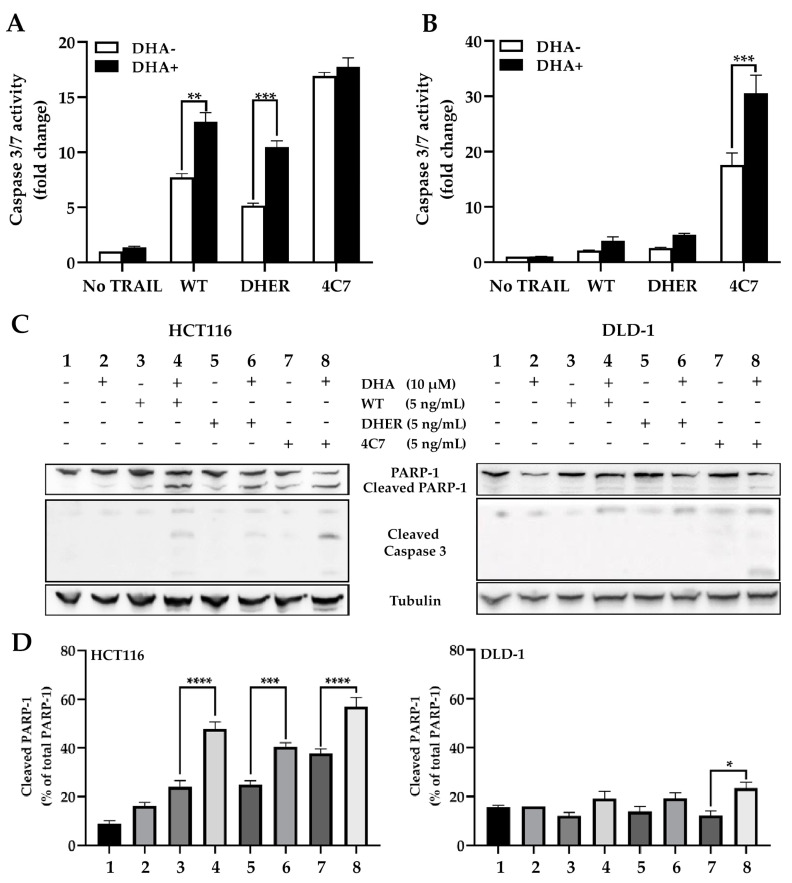
DHA activates caspase-mediated cell death differently through the two receptors. The caspase-3/7 activity was performed on HCT116 (**A**) and DLD-1 (**B**), and the luminescence was measured after 1 h reagent incubation. Data shown are mean ± SEM from two independent experiments performed in triplicate. (**C**) Western blot analysis of full-length PARP-1, cleaved PARP-1, and cleaved caspase-3 using γ-Tubulin as the loading control. (**D**) The cleaved PARP-1 relative to total PARP-1 was calculated based on integrated density relative to γ-Tubulin. Figure S1A,B show the original Western blot. All cells were treated with 10 μM DHA for 30 min, followed with/without 5 ng/mL rhTRAIL WT, DHER, or 4C7 for 12 h to check the caspase-3/7 activity, 24 h for Western blot. *p* values were analyzed by two-way ANOVA with Sidak’s multiple comparisons test in Graphpad Prism version 8.0. * 0.01 < *p* ≤ 0.05, ** 0.001 < *p* ≤ 0.01, *** 0.0001 < *p* ≤ 0.001, **** *p* ≤ 0.0001.

The caspase-3/7 activity was analyzed in single and combination treatments to comprehend the influence of DHA on TRAIL-induced apoptosis. It was found that, compared with DHA untreated cells, an increase in caspase-3/7 activity was observed in all TRAIL treated groups in HCT116. Apparent changes were shown between the single WT group (7.7-fold) and DHA and WT group (12.8-fold), single DHER group (5-fold), and DHA and DHER group (10.5-fold). The addition of DHA has a significant effect on the WT or DHER treated groups, but no additional effect was observed in the 4C7 group (Figure 4A), which can be explained by the already very high activity of 4C7 alone. In DLD-1, a similar trend was observed, apart from a substantial increase in the caspase-3/7 activity in the presence of DHA in the 4C7 group (Figure 4B). This result corresponds to Figure 3B, showing that DLD-1 is highly sensitive to 4C7 but resistant to DHER. Moreover, the difference in the combination treatment of DHER in these two cell lines is far more interesting in that DHA overcomes the DHER resistance in HCT116. Furthermore, the Western blot analysis of PARP-1, cleaved PARP-1, and cleaved caspase-3, which are the hallmarks of cell death, shows a similar result (Figure 4C,D). It indicates that DHA induces cell death through caspase-3 activation, which enhances TRAIL-induced apoptosis, but the influence via the two death receptors may be varying in different cell lines.

### 2.3. ATS and DHA Induce the Gene Transcription of Death Receptors in Colon Cancer Cells

**Figure 5:**
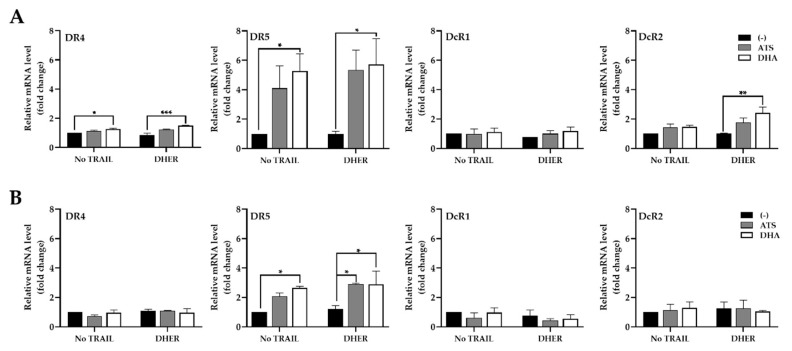
ATS and DHA increase the gene expression of DR5 in HCT116 and DLD-1. Relative mRNA levels of DR4, DR5, DcR1, and DcR2 in HCT116 (**A**) and DLD-1 (**B**). The total RNA was extracted from the cells treated with 10 μM ATS/DHA for 30 min, followed with/without 5 ng/mL DHER for 24 h. Relative gene expression of the receptors (normalized to GAPDH) was analyzed by qRT-PCR and transduced with control. Data shown are mean ± SEM from two independent experiments performed in triplicate. *p* values were analyzed by two-way ANOVA with Dunnett’s multiple comparisons test in Graphpad Prism version 8.0. * 0.01 < *p* ≤ 0.05, ** 0.001 < *p* ≤ 0.01, *** 0.0001 < *p* ≤ 0.001.

Our previous data showed that DHA in combination with rhTRAIL WT or variants stimulates relatively significant differences in caspase-3/7 activity in HCT116 and DLD-1. It is tempting to investigate the role of the different TRAIL receptors by looking at their gene transcription levels. It appears specifically that the DR5 transcription level is remarkably increased in the presence of DHA (Figure 5A), whereas the transcription of the genes encoding DR4 and DcR2 shows a small uptick in HCT116. The expression of DcR1 remains stable in all treatments in both HCT116 and DLD-1. A slight increase in DR5 mRNA level is observed in DLD-1, but the fold change is much lower than the one in HCT116 (Figure 5B). Moreover, the DR4 transcription level remains steady in all treatments, which indicates that ATS/DHA does not affect the DR4 expression in DLD-1 (Figure 5B). Finally, the combination treatment with DHER does not explain major differences in transcription levels from all the receptors compared with single-drug treatment in both cell lines.

### 2.4. ATS and DHA Increase the Death Receptors Expression on Colon Cancer Cell Surface

**Figure 6:**
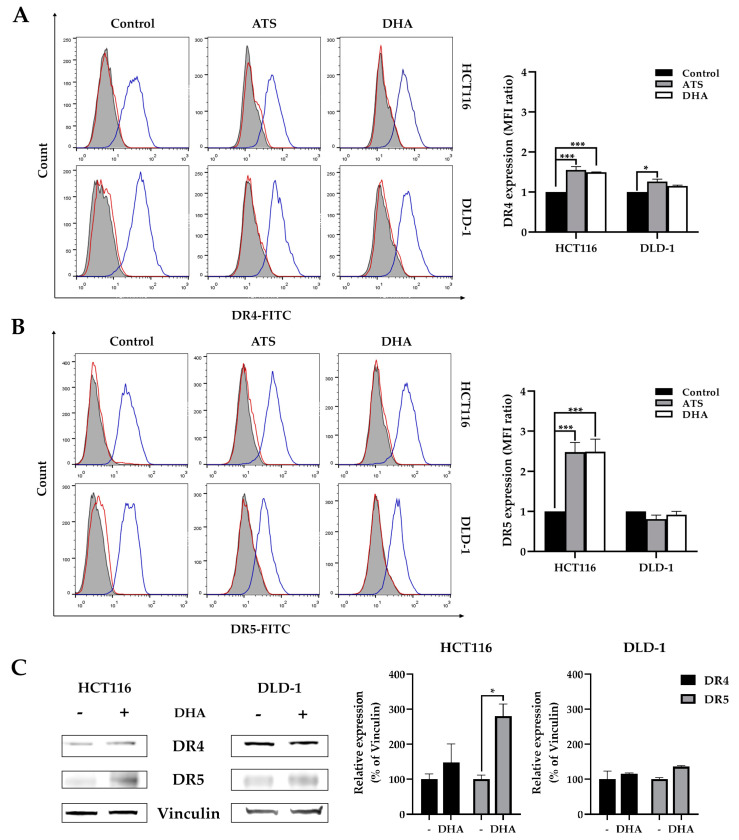
Artemisinin derivatives upregulate death receptor expression mainly in HCT116. HCT116 and DLD-1 were treated with 10 μM ATS/DHA for 24 h and harvested to analyze cell surface DR4 (**A**) and DR5 (**B**) expression by immunofluorescent staining followed with flow cytometry. The left panel shows the histograms of death receptors, and the right figures show the mean fluorescence intensity (MFI) ratio relative to IgG isotype (red open line). The filled grey peaks represent unstained cells, and blue open lines represent DR4 or DR5, respectively. (**C**) DR4 and DR5 expression in 10 μM DHA treated HCT116 and DLD-1 using vinculin as the loading control. Figure S1C shows the original Western blot. Data shown are mean ± SEM from two independent experiments. *p* values were analyzed by two-way ANOVA with Tukey’s multiple comparisons test in Graphpad Prism version 8.0. * 0.01 < *p* ≤ 0.05, *** 0.0001 < *p* ≤ 0.001.

As the transcription level of the DR5 gene was increased, we investigated whether ATS and DHA also enhance the expression of death receptors on the cell surface. We focused on the effects of artemisinin derivatives to DR4 and DR5 in HCT116 and DLD-1 cell lines using flow cytometry analysis (Figure 6A,B). The highest increase (150%) was detected for DR5, and a smaller increase (50%) was seen for DR4 after treatment with ATS/DHA in HCT116 cells. While, in DLD-1, the expression of both DR4 and DR5 remains unchanged, it seems that the increasing of DR5 transcription level is insufficient to be observed on the cell surface. These changes in DR4 and DR5 expression may form the basis of the enhanced sensitivity of colon cancer cells to TRAIL-induced apoptosis. It should be noted that we do detect a small increase in DR4 expression using FACS analysis, which is in line with the earlier tendency in the increase of DR4 mRNA levels in Figure 5A. The expression of DR4 and DR5 with DHA in absence were also analyzed by Western blot, as shown in Figure 6C, which shows the same trend observed in Figure 6A,B.

### 2.5. DHA Increases the Gene Transcription of P53 and Its Downstream Targets

**Figure 7:**
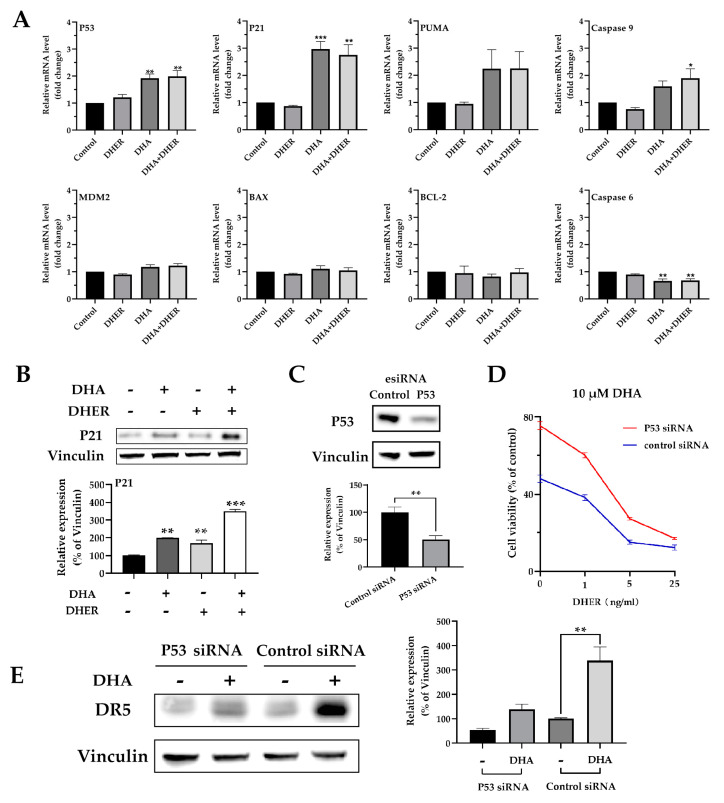
DHA influences the p53 pathway in HCT116. (**A**) Relative mRNA levels of P53, P21, PUMA, caspase-9, MDM2, BAX, BCL-2, and caspase-6 in HCT116. (**B**) Western blot analysis of P21 (upper) and the integrated density of P21 relative to vinculin (bottom). *p* values were compared with control cells in each group. The cells were treated with 10 μM DHA for 30 min, followed with/without 5 ng/mL DHER for 24 h. (**C**) The Western blot analysis of P53 in HCT116 after 72 h control siRNA or P53 siRNA transfection. HCT116 cells were transfected with control siRNA or P53 siRNA for 48 h, followed with 24 h treatment. Then, the MTS (**D**) or Western blot of DR5 (**E**) were performed. Figure S3 shows the original Western blot. Relative gene expression (normalized to GAPDH) was analyzed by qRT-PCR and transduced with control. *p* values were analyzed by two-way ANOVA with Dunnett’s multiple comparisons test in Graphpad Prism version 8.0. * 0.01 < *p* ≤ 0.05, ** 0.001 < *p* ≤ 0.01, *** 0.0001 < *p* ≤ 0.001.

To gain insight into the regulating routine from artemisinin to death receptors, qPCR assay was performed, and the relative mRNA levels of P53, P21, PUMA, caspase-9, MDM2, BAX, BCL-2, and caspase-6 were analyzed in HCT116 (Figure 7A) and DLD-1 (Figure S2A). DHA significantly enhances the transcription level of P53 and its downstream target P21, PUMA, and caspase-9 in HCT116. As a negative regulator of P53, MDM2 shows a slight increase in DHA treatment. Meanwhile, the upregulation of P53 shows no influence on BAX and BCL-2 but decreases caspase-6. In contrast to the wild type status in HCT116, P53 contains a single mutation, Ser^241^Phe, in the DNA binding domain in DLD-1. We only observed a slight increase in the transcription levels of P53, P21, and PUMA under DHA treatment in DLD-1 (but not significant); meanwhile, no effects were observed for the rest of the target genes. The Western blot analysis of P21 in HCT116 (Figure 7B) corresponds to the qPCR results, while less P21 is detected in all treatments in DLD-1 (Figure S2B). 

The P53 knockdown assay was performed to confirm the role of p53 and DR5 in DHA treated HCT116. Western blot analysis showed a 50% (*p* < 0.01) reduction in P53 expression in the knockdown cells (Figure 7C). This knockdown results in an increase in cell viability (25–30%) in HCT116 after 10 μM DHA treatment. The protective effect was also observed in the combination treatment of DHA with DHER (Figure 7D). After being treated with DHA, DR5 expression is significantly increased by 200% in control cells, whereas a less dramatic increase (100%) is observed in P53 knockdown cells (Figure 7E). These findings suggest a complementary role for p53 in the upregulation of DR5 in response to treatment with artemisinin derivatives.

### 2.6. DHA Improves TRAIL-Induced Apoptosis in the 3D Spheroid Model

**Figure 8:**
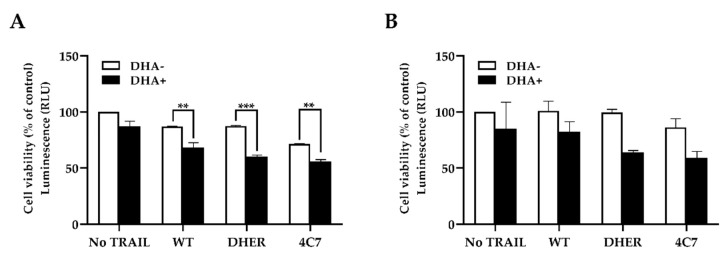
DHA sensitizes HCT116 and DLD-1 spheroids to TRAIL-induced apoptosis. The 3D spheroids of HCT116 (**A**) and DLD-1 (**B**) were constructed in ultra-low attachment 96-well plates after 72 h incubation. Then, the spheroids were treated with 10 μM DHA for 30 min, followed with/without 25 ng/mL rhTRAIL WT, DHER, or 4C7 for 24 h. The cell viability of the spheroids was determined after 25 min incubation with CellTiter-Glo^®^ 3D Reagent. Data shown are mean ± SEM from three independent experiments performed in triplicate. *p* values were analyzed by two-way ANOVA with Sidak’s multiple comparisons test with Graphpad Prism version 8.0. ** 0.001 < *p* ≤ 0.01, *** 0.0001 < *p* ≤ 0.001.

To further confirm the efficacy of DHA for colon cancer cells, we used 3D spheroids grown from HCT116 and DLD-1 cells to mimic the actual tumor microenvironment. Cells grown in 3D are considered as non-vascularized tumor models that better reflect the 3D cell–cell, cell–matrix interactions, and the biochemical environment of in vivo tumor mass compared to 2D cell models [29]. We incubated the 3D spheroids with single or combination treatments for 24 h after the spheroids were established (Figure S4A,B). The spheroids were subsequently incubated with CellTiter-Glo^®^ 3D Reagent to analyze the cell viability. In both HCT116 and DLD-1 spheroids, DHA pretreating decreases the cell viability in all TRAIL WT or variant treated groups (black bar) compared with those without DHA (white bar) (Figure 8A,B). Interestingly, the spheroids cell death is the additive of two drugs in WT and 4C7 groups, but DHER develops evident efficacy after being treated with DHA in HCT116, which shows the same result as the MTS assay (Figure 3A). This means that DHA overcomes DHER resistance and induces more cell death in the 3D HCT116 cell model.

## 3. Discussion

For clinical therapy, TRAIL and the death receptor agonistic antibodies are under phase II clinical trials in colorectal cancer treatment due to TRAIL’s harmfulness to cancer cells compared with healthy cells [28,30]. However, the recombinant human TRAIL dulanermin already showed reduced efficacy in phase I clinical trial as a result of low bioavailability and decoy receptor binding [31,32]. Therefore, in this project, DR4-specific TRAIL variant 4C7 and DR5-specific TRAIL variant DHER were used to reduce the influence of decoy receptors. Furthermore, some human agonistic monoclonal antibodies were investigated in phase I and II clinical trials, e.g., mapatumumab targets DR4 [33], conatumumab [34,35], and drozitumab [36,37] target DR5. All of them were well tolerated in patients but showed no or little benefit in progression-free survival in comparison with the control treatment. These consequences were mainly from the resistance to TRAIL-induced apoptosis in colorectal cancer, which is associated with various stages of the TRAIL signaling pathway.

The mutation or epigenetic change in the *p53* and *K-Ras* status has substantial responsibility in regulating death receptor expression [38,39]. In addition, the abnormal downregulation or inactivation of caspase-8, the inhibition of c-FLIP, or other anti-apoptotic proteins also decrease the intracellular signaling of death receptors [40,41]. To deal with the resistance of TRAIL-induced apoptosis, artemisinin derivatives were chosen to pretreat the colon cancer cell lines. As the most potent and rapidly acting antimalarial agents, artemisinin and its derivatives have been well tested in human bodies—better still, the usage and adverse reactions have been fully established. At the same time, multiple studies show artemisinin’s anti-cancer properties and the possibility of overcoming TRAIL resistance in different cancer cell lines [20,21,42]. In this research, we found that ATS/DHA has even better anti-cancer activity compared to ART. By producing ROS in cancer cells, ATS/DHA releases the oxidative stress leading to DNA damage, p53 activation, apoptotic, or non-apoptotic cell death [43,44]. 

Both HCT116 and DLD-1 are resistant to DHER but more susceptible to TRAIL WT and 4C7. With the pretreatment of ATS/DHA, the cell viability is remarkably reduced with the increasing DHER in HCT116, while a minor effect is observed in DLD-1. We proved that cell viability is reduced via cell apoptosis after the double treatment with DHA and DHER by annexin V/PI assay and caspase activity assay. Thus, ATS and DHA overcome the resistance to DHER-induced apoptosis in HCT116 and do so slightly in DLD-1 (Figure 3E). We also confirmed that ATS/DHA induces the transcription level of DR5 in both cell lines (Figure 5). However, in DLD-1 cells, the slight increase in DR5 gene expression is insufficient to increase its expression on the cell surface (Figure 6B). Therefore, DLD-1 is less sensitive to the combination treatment of ATS/DHA with DHER. 

The *p53* gene status is wild type in HCT116, whereas it contains a single mutation, Ser^241^Phe, in the DNA-binding domain in DLD-1. As a transcription factor, P53 binds the target gene through a p53 binding site (P53BS) to regulate downstream gene expression. DR4, DR5, DcR1, and DcR2 might be directly regulated by P53 due to the P53BS identified in their first introns [45,46]. In our study, DHA significantly increases the transcription level of P53 (Figure 7A), with the most pronounced enhancement of DR5 expression in HCT116 (Figure 5A). With the P53 knockdown in HCT116, we prove that DHA induces DR5 expression through regulating P53 (Figure 7D,E). For DLD-1, even with a similar influence from DHA on P53 expression, the mutation in its DNA-binding site leads to fewer changes in the transcription levels of death receptors and the downstream targets (Figure 5B and Figure S2A). It is also reported that the P53 with a mutation on its DNA binding site rarely transactivates the DR5 compared with WT P53 [47,48]. Bringing together all the changes in HCT116 and DLD-1 under artemisinin derivative treatment, ROS induces the expression of WT P53 and further upregulates DR5 to overcome DHER resistance in HCT116. Meanwhile, with the mutated P53 in DLD-1, only a minor effect on the expression of death receptors and cell death was observed.

Finally, using the 3D spheroids culture of HCT116 and DLD-1 mimicking the actual tumor microenvironment of colon carcinoma, we found that the combination of ATS/DHA with DHER induces more cell death in HCT116. It is confirmed that ATS/DHA induces the expression of DR5 in HCT116, which further stimulates the cell line to DHER-induced apoptosis. Our results have proposed a new perspective for killing cancer cells with fewer DR5 on the cell surface.

## 4. Materials and Methods

### 4.1. Cell Lines and Reagents

Human colorectal cancer cell line COLO205, DLD-1, HCT116 were obtained from American Type Culture Collection (ATCC, Wesel, Germany) and cultured in RPMI1640 medium supplemented with 10% fetal bovine serum (FBS), 100 units/mL penicillin, and 100 µg/mL streptomycin in a humidified incubator at 37 °C and 5% CO_2_. All materials mentioned above were obtained from Thermofisher Scientific (Landsmeer, The Netherlands). The wild type (WT) TRAIL, DR4-specific TRAIL variant 4C7, and DR5-specific TRAIL variant DHER were produced as described previously [26,27]. Artemisinin (ART; Sigma-Aldrich, St. Louis, MO, USA), artesunate (ATS; Sigma-Aldrich, St. Louis, MO, USA) and dihydroartemisinin (DHA; Adooq Bioscience, Irvine, CA, USA) were prepared by dissolving in dimethyl sulfoxide (DMSO). The final concentration of DMSO was less than 1% in all experiments.

### 4.2. Cell Viability Assay

Cells were seeded in triplicate in 96-well plates at cell densities of 10,000 cells/well for DLD-1, 5000 cells/well for COLO205, and HCT116 in 100 µL medium for 24h. For individual treatment, cells were treated with ART, ATS/DHA in concentrations of 0, 1, 5, 10, 50, and 100 µM or TRAIL WT in concentrations of 0, 1, 10, and 100 ng/mL, with a final volume of 150 µl/well. For combination treatment, cells were treated with 10 µM ATS/DHA for DLD-1 and HCT116 for 30 min and 1–25 ng/mL rhTRAIL WT, 4C7, or DHER were added. After 24 h incubation, cells were incubated with 20 uL/well MTS reagent according to the manufacturer’s instructions (Promega, Leiden, The Netherlands). The absorbance was recorded at 490 nm on a microplate reader (Omega, BMG LABTECH GmbH, Ortenberg, Germany).

### 4.3. Apoptotic Assay

Cell apoptosis detection was performed using eBioscience™ Annexin V-FITC/PI Apoptosis Detection Kit (Thermo Fisher Scientific, Carlsbad, CA, USA) with FACS Calibur flow cytometer (BD Biosciences). After 16 h treatment, cells were harvested and washed with the binding buffer from the kit. Then, the cells were resuspended in 100 μL binding buffer containing 3 μL annexin V and incubated for 15 min at RT. After the washing step, 3 μL of propidium iodide was added into 200 μL cell suspension. Cells were analyzed by flow cytometry, and 10,000 events were recorded for each FCM analysis. 

### 4.4. Caspase 3/7 Activity Assay

HCT116 and DLD-1 cells were seeded into a white-walled 96-well plate and cultured overnight. Cells were pretreated with 10 µM DHA for 30 min, followed by 5 ng/mL rhTRAIL, 4C7, or DHER for 12 h. The Caspase-Glo^®^ 3/7 Reagent (Promega Corporation, Madison, WI, USA) was equilibrated to RT, and 50 µl of the reagent was added to each well. The luminescence was collected after the plate was gently mixed and incubated at RT for 1 h.

### 4.5. Western Blot Analysis

Cells were harvested and lysed in RIPA buffer with Complete Protease Inhibitor Cocktail, EDTA-Free (Roche, Basel, Switzerland) inside. Protein concentrations were determined using the Pierce BCA Protein Assay Kit (Thermo Fisher Scientific, Rockford, IL, USA) and Western blot analyses were performed as previously described [28]. PARP-1, caspase-3 (Cell Signaling Technology, Leiden, The Netherlands), DR4 (Novus Biologicals, Abingdon, UK), DR5, P21, P53 (abcam, Cambridge, UK) primary antibodies were used. Anti-γ-Tubulin or anti-vinculin (Sigma Aldrich, St Louis, MO, USA) was used for confirmation of equal loading of proteins. Polyclonal rabbit anti-mouse immunoglobulins/HRP and polyclonal goat anti-rabbit immunoglobulins/HRP (Dako, Glostrup, Denmark) were used as the secondary antibody. 

### 4.6. RNA Extraction and Quantitative Reverse Transcription PCR

Total RNA was extracted using the Maxwell 16 LEV simplyRNA tissue kit according to the manufacturer’s instructions (Promega, Madison, WI, USA) and the concentration was measured by NanoDrop (Thermo Fisher Scientific, Carlsbad, CA, USA). cDNA was synthesized from 500 ng RNA using Reverse Transcription Kit (Promega, Madison, WI, USA). The expression of TRAIL receptors, P53, P21, PUMA, caspase-9, MDM2, BAX, BCL-2, caspase-6 was studied by SensiMix SYBRkit (Bioline, Taunton, MA, USA) using qRT-PCR using QuantStudio™ 7 Flex System (Thermo Fisher Scientific, Rockford, IL, USA). The expression levels were calculated relative to GAPDH, and the primers are listed in Table S1. 

### 4.7. Flow Cytometry of Death Receptor Expression

Cells were seeded in 6-well plates at a density of 500,000 cells/well overnight. The next day, 10 μM ATS/DHA was added to HCT116 and DLD-1. After 24 h, cells were harvested and washed with FACS buffer (PBS with 2% FBS). 

Presence of DR4 and DR5 receptors on the cell surface of HCT116 and DLD-1 human colorectal cancer cells was determined by FACS. Cells were collected and resuspended in FACS buffer. Then, they were washed and incubated with anti-DR4 antibody (Abcam, Cambridge, UK) or anti-DR5 antibody (Exbio, Praha, Czech Republic). After washing again, the fluorescein (FITC) conjugated donkey anti-mouse IgG (Jackson ImmunoResearch, Cambridge, UK) was incubated with cells on ice for 1 h. Isotype control staining was performed with mouse IgG (Dako, Glostrup, Denmark). Death receptor expression was measured using the FACS Calibur flow cytometer (BD Biosciences), and data were analyzed by FlowJo v10 (FlowJo, LCC, Oregon, OR, USA). 

### 4.8. P53 Knockdown by Using siRNA

For P53 knockdown, HCT116 cells were transfected using Lipofectamine™ 2000 Transfection Reagent (Thermo Fisher Scientific, Waltham, MA, USA). Cells were seeded at 200,000 cells/well in 6-well plates and cultured for 24 h. Then, the cells were transfected with a predesigned pool of *TP53* human esiRNA or control esiRNA (Sigma-Aldrich, Zwijndrecht, The Netherlands) at a final concentration of 15 pmol/well. After 48 h incubation, the cells were treated with/without 10 µM of DHA for 24 h and then collected for Western blot. The *TP53* siRNA interfering assay was performed in triplicate.

### 4.9. 3D Spheroid Construction and Cell Variability

Cells from colorectal cell lines HCT116 and DLD-1 were seeded (2000 cells/well) in ultra-low attachment 96-well plates (Corning Incorporated, Kennebunk, ME, USA). The plate was spun down for 5 min at 1000 rpm and cultured for 3 days. After culturing, the cells were pretreated with 10 µM ATS/DHA for 30 min, and 5 ng/mL TRAIL WT, DHER, or 4C7 was added. After 24 h treatment, an equal volume of CellTiter-Glo^®^ 3D Reagent (Promega Corporation, Madison, WI, USA) was added to each well according to the technical manual, and the luminescence was recorded with a Synergy™ H1 plate reader (BioTek Instruments, Winooski, VT, USA) after 25 min incubation at room temperature (RT). Pictures were taken with the Leica Microscope type EC3 (Leica Microsystems (Heerbrugg, Switzerland) after being treated for 24 h. 

### 4.10. Statistical Analysis

Data were presented as a mean ± standard deviation (SD) from three measurements in one experiment or a mean ± standard error of the mean (SEM) from independent experiments. Multiple comparisons test was analyzed by two-way ANOVA with GraphPad Prism version 8.0 (GraphPad Software, San Diego, CA, USA).

## 5. Conclusions

Our study shows that artemisinin derivatives ATS and DHA sensitize HCT116 to DR5-specific TRAIL-induced apoptosis but show weaker effects on DLD-1. With further investigation, we found that the difference is mainly due to the P53 status in these two cell lines. In HCT116, WT P53 makes it more sensitive to ATS/DHA and leads to an increase in DR5 on the cell surface. At the same time, this result is also proven by the 3D spheroid model, which shows that the combination treatment of ATS/DHA with DR5-specific TRAIL variant DHER is a potential therapy to kill colon cancer cells.

## Supplementary Materials

The following are available online at https://www.mdpi.com/2072-6694/12/9/2514/s1, Table S1: List of primers used for qRT-PCR, Figure S1: The whole blot showing all the bands with molecular weight markers on the Western as the supplementary for Figure 4 and Figure 6, Figure S2: The influence of DHA on p53 pathway in DLD-1, Figure S3: The whole blot showing all the bands with molecular weight markers on the Western as the supplementary for Figure 7, Figure S4: The 3D spheroids morphology changes under DHA treatment in HCT116 and DLD-1.
