# Supplementary material for: Artemisinin Derivatives Stimulate DR5-Specific TRAIL-Induced Apoptosis by Regulating Wildtype P53
Source: Cancers (Basel). 2020 Sep 4;12(9):2514. doi: 10.3390/cancers12092514 (PMC7563660; doi:10.3390/cancers12092514)
Supplement: Supplementary file 1 [file cancers-12-02514-s001.pdf]

cancers

MDPI

Artemisinin Derivatives Stimulate DR5-Specific TRAIL-Induced Apoptosis by Regulating Wild Type P53

Xinyu Zhou, Sietske N. Zijlstra, Abel Soto-Gamez, Rita Setroikromo and Wim J. Quax

Table S1. List of primers used for qRT-PCR.

Name	Strand	Sequence
GAPDH	F	5'-ACCCAGAAGACTGTG-3'
	R	5'-TCTAGACGGCAGGTC-3'
DR4	F	5'-CAGAACGTCCTGGAGCCTGTAAC-3'
	R	5'-ATGTCCATTGCCTGATTCTTTGTG-3'
DR5	F	5'-TGCAGCCGTAGTCTTGATTG-3'
	R	5'-GCACCAAGTCTGCAAAGTCA-3'
DcR1	F	5'-CACCAACGCTTCCAACAATGAACC-3'
	R	5'-TCCGGAAGGTGCCTTCTTTACACT-3'
DcR2	F	5'-CTTTTCCGGCGGCGTTCATGTCCTTC-3'
	R	5'-GTTTCTTCCAGGCTGCTTCCCTTTGTAG-3'
P53	F	5′-GCCCCTCCTCAGCATCTTAT-3′
	R	5′-AAAGCTGTTCCGTCCCAGTA-3′
P21	F	5′-CTGGGGATGTCCGTCAGAAC-3′
	R	5′-CATTAGCGCATCACAGTCGC-3′
PUMA	F	5′-CTCGGTGCTCCTTCACTCTG-3′
	R	5′-AGGCTAGTGGTCACGTTTGG-3′
Caspase 9	F	5′-AAAGTTGTCGAAGCCAACCC-3′
	R	5′-GACTCACGGCAGAAGTTCAC-3′
MDM2	F	5′-CAGCAGGAATCATCGGACTCA-3′
	R	5′-AGGTCCTTTTGATCACTCCCAC-3′
BAX	F	5′-AACATGGAGCTGCAGAGGAT-3′
	R	5′-CCAATGTCCAGCCCATGATG-3′
BCL-2	F	5′-TTCTTTGAGTTCGGTGGGGT-3′
	R	5′-CTTCAGAGACAGCCAGGAGA-3′
Caspase 6	F	5′-ATGGCGAAGGCAAATCACATTT-3′
	R	5′-GTGCTGGTTTCCCCGACAT-3′

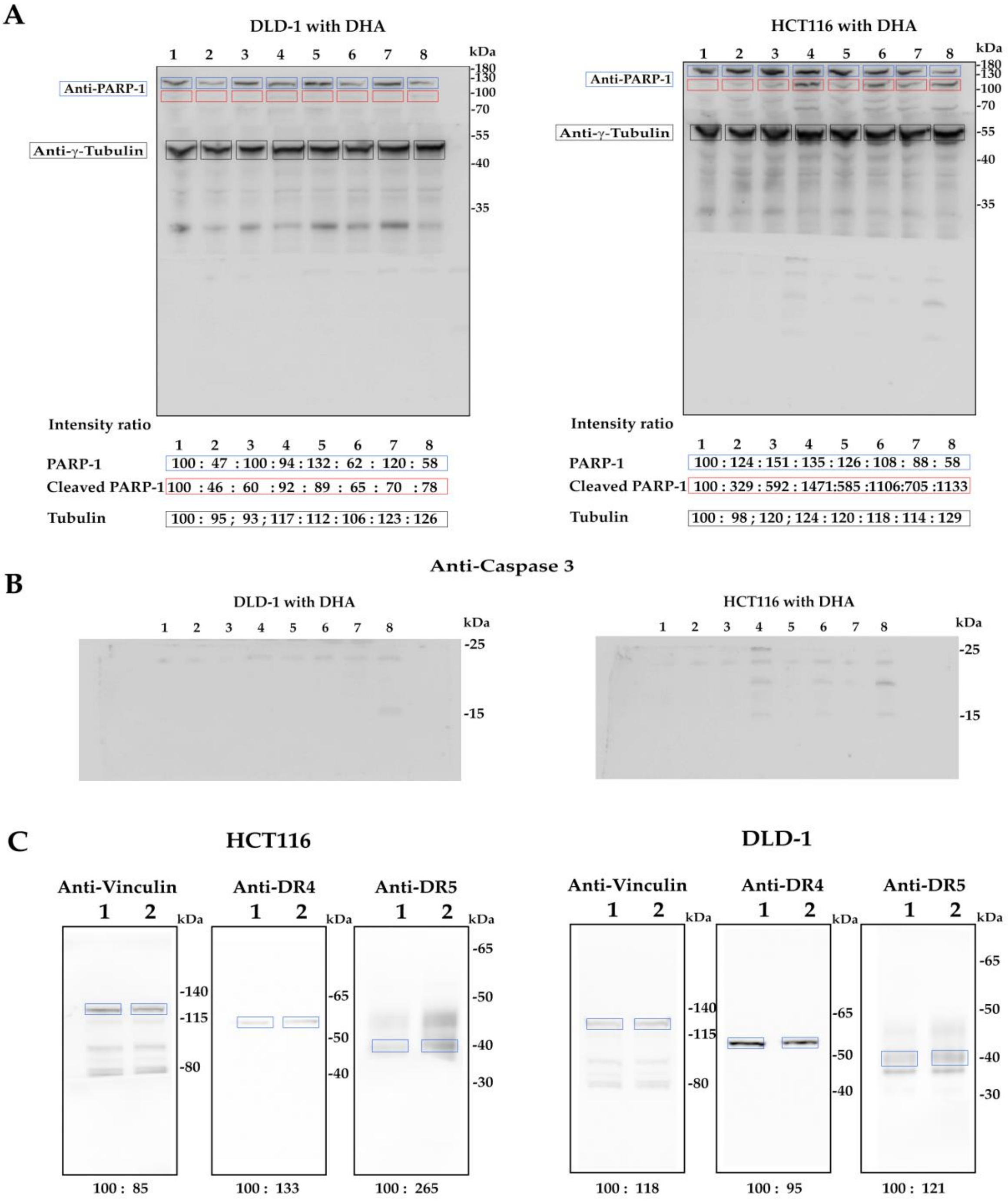

Figure S1. The whole blot showing all the bands with molecular weight markers on the Western as the supplementary for Figure 4 and 6. (A) and (B) for Figure 4C; (C) for Figure 6C.

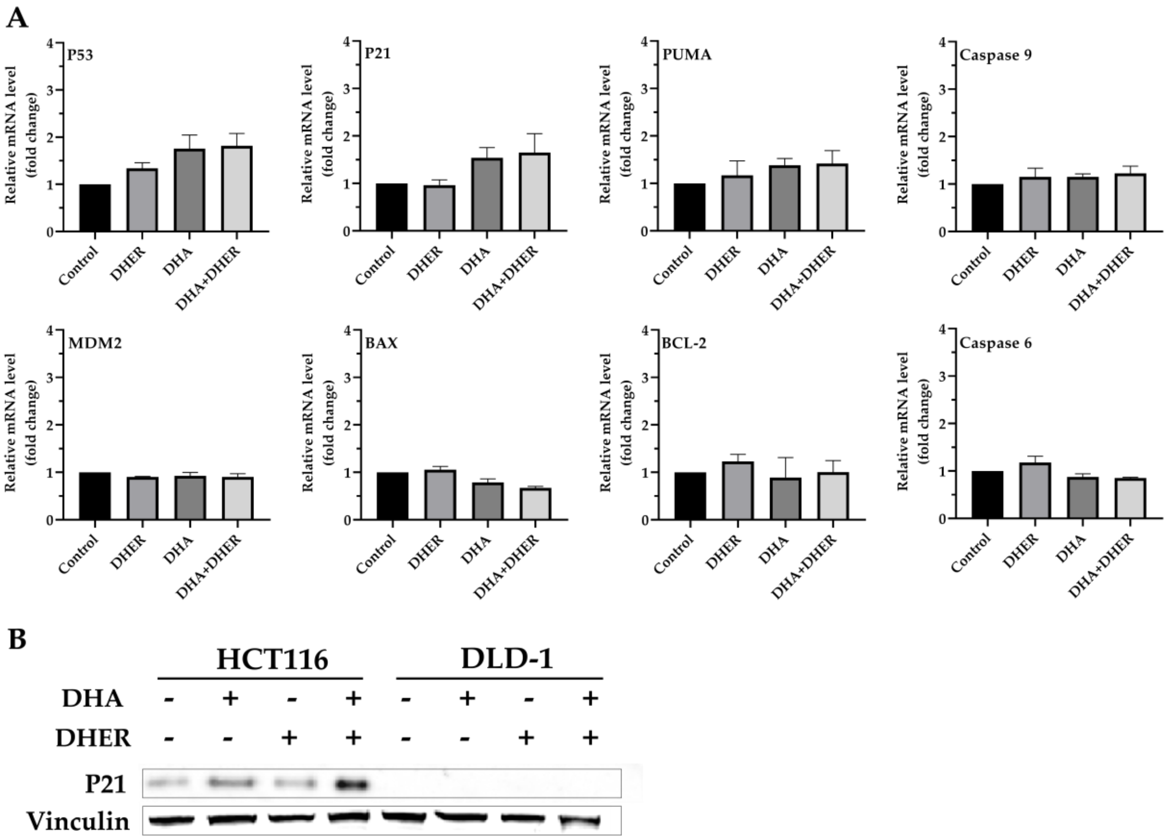

Figure S2. The influence of DHA on p53 pathway in DLD-1. (A) Relative mRNA levels of P53, P21, PUMA, Caspase 9, MDM2, BAX, BCL-2, Caspase 6 in DLD-1. (B) The western blot of P21 in HCT116 and DLD-1 using Vinculin as the loading control. The cells were treated with 10 μM DHA for 30 min followed with/without 5 ng/ml DHER for 24 h. Relative gene expression (normalized to GAPDH) was analyzed by qRT-PCR and transduced with control. Data shown are mean ± SEM from three independent experiments performed in triplicate. p values were compared with control cells in each group and analyzed by two-way ANOVA with Dunnett multiple comparisons test in Graphpad Prism version 8.0.

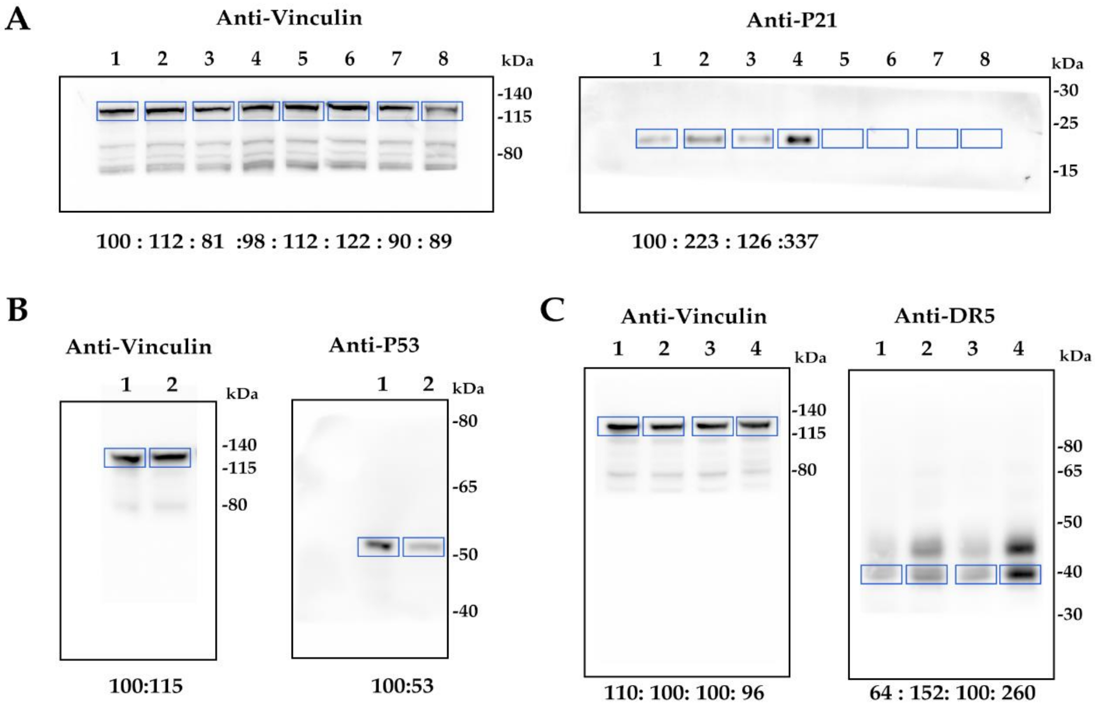

Figure S3. The whole blot showing all the bands with molecular weight markers on the Western as the supplementary for Figure 7. (A) for Figure 7B; (B) for Figure 7C; (C) for Figure 7E

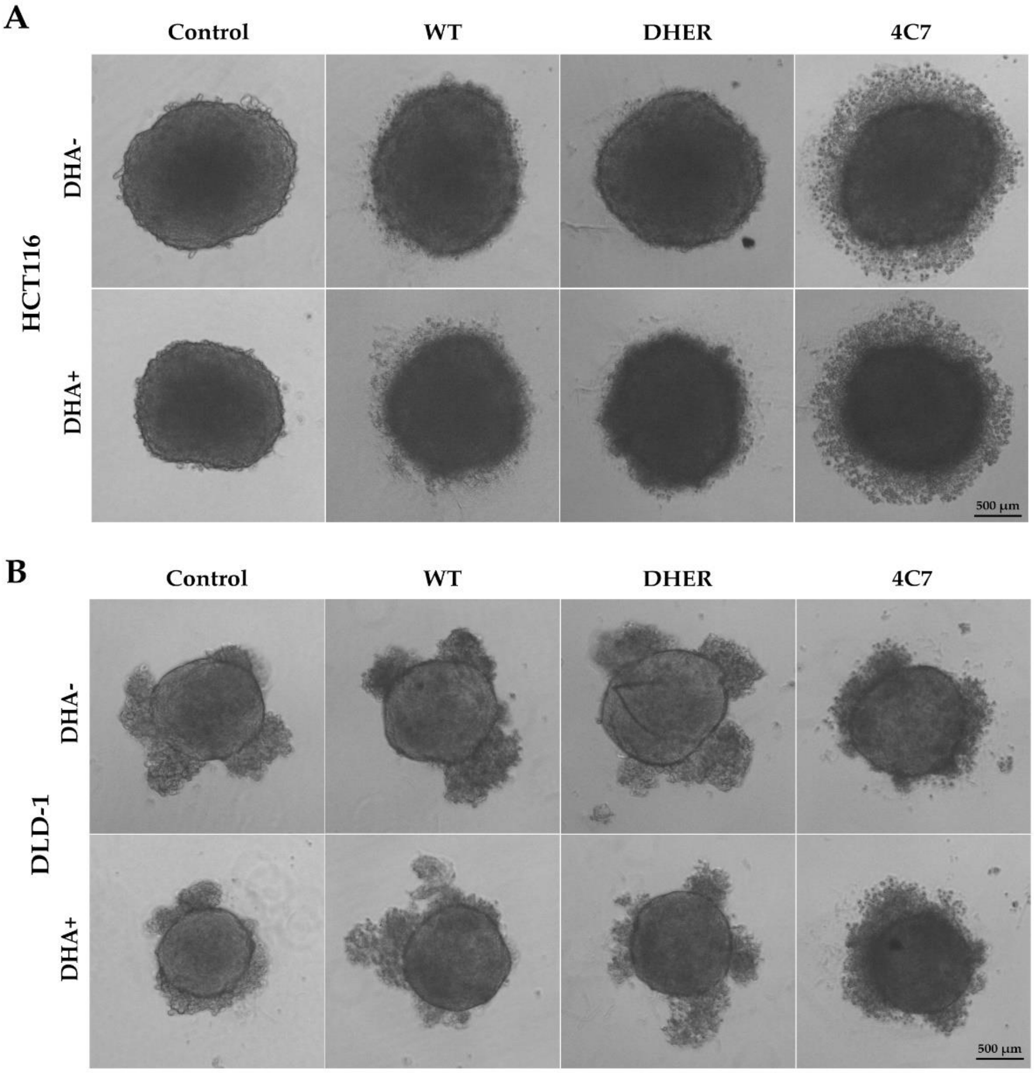

Figure S4. The 3D spheroids morphology changes under DHA treatment in HCT116 (A) and DLD-1 (B). The 3D spheroids of HCT116 and DLD-1 were constructed in ultra-low attachment 96-well plates after 72 h incubation. Then the spheroids were treated with 10 μM DHA for 30 min followed with/without 25 ng/ml rhTRAIL WT, DHER or 4C7 for 24 h. Morphology changes were observed under an inverted light microscope with 40× magnification.

© 2020 by the authors. Submitted for possible open access publication under the terms and conditions of the Creative Commons Attribution (CC BY) license (http://creativecommons.org/licenses/by/4.0/).
